# Cancer of the Breast

**Published:** 1890-02

**Authors:** 


### CANCER OF THE BREAST.

Dr. A. G. Gerster, of New York, recently said in a clinical lecture: In operating on late cases of cancer, never be too sanguine of success, nor urge your patient too earnestly to submit to an operation. There are, however, times when urgency is of the very greatest importance, and it is your duty as surgeons to exercise pressure at this time. Let me tell you, and I will speak plainly now, that grave sins of omission are to be laid at the door of the general practitioner in this regard, and I say this with the utmost respect for the general practitioner, who is a man of hard common sense, and who knows what he is about.

The time when this urgency is demanded of the general practitioner *is when the patient presents at his office for the first time with a little nodule on her breast and complaining of pain running down her arm.* She is a woman of middle age, who has perhaps nursed many children. Under such circumstances what does the general practitioner do? Does he warn his patient of the danger of her life and say to her that if this little nodule is not taken out at once her chances for recovery are very bad? Does the general practitioner do this? No; he simply prescribes a belladonna plaster, or some other equally useless remedy. He knows beforehand it is a useless remedy and still he prescribes it.

When a woman presents herself to you with a tumor on the breast, she ought at once to be operated on, for it must be borne in mind that ninety-five per cent. of the tumors of the female breast are of cancerous origin. I will, however, qualify that statement, for a woman may possibly have syphilitic mastitis, or chronic suppurative mastitis.

If you suspect a syphilitic mastitis, by giving the patient thirty grains of iodide of potass. three times a day, the tumor will rapidly disappear, if it be due to syphilis, and there will be a decided improvement in the appearance of the patient.

If, on the other hand, it be a cyst, you can always puncture it and evacuate its contents. In a woman past her menopause, cases which are not cancerous may very soon develop into cancer.

When a woman presents herself with a tumor in the breast, who has lost flesh and strength, and who complains of a running pain in the arm and breast, I would advise that such a woman be put on iodide of potass. for a few days, and if that does no good, then anæsthetize that patient and make an opening in the tumor. If it be a cyst simply, evacuate and excise it, sew up the wound, and the cyst is completely cured. There are few women who will not submit to such an operation. Before the advent of the antiseptic mode of treating wounds, 30 to 33 per cent. of women operated upon for tumor of the breast died as a result of the operation. At that time it was a serious matter to propose amputation of the breast. To-day, as a result of the operation, we do not lose one per cent.—*Practice.*

